# Tuberculin skin test and QuantiFERON-Gold In Tube assay for diagnosis of latent TB infection among household contacts of pulmonary TB patients in high TB burden setting

**DOI:** 10.1371/journal.pone.0199360

**Published:** 2018-08-01

**Authors:** Padmapriyadarsini Chandrasekaran, Vidya Mave, Kannan Thiruvengadam, Nikhil Gupte, Shri Vijay Bala Yogendra Shivakumar, Luke Elizabeth Hanna, Vandana Kulkarni, Dileep Kadam, Kavitha Dhanasekaran, Mandar Paradkar, Beena Thomas, Rewa Kohli, Chandrakumar Dolla, Renu Bharadwaj, Gomathi Narayan Sivaramakrishnan, Neeta Pradhan, Akshay Gupte, Lakshmi Murali, Chhaya Valvi, Soumya Swaminathan, Amita Gupta, for the CTRIUMPH Study Team

**Affiliations:** 1 Department of Clinical Research, National Institute for Research in Tuberculosis, Chennai, India; 2 Johns Hopkins University School of Medicine, Baltimore, United States of America; 3 Byramjee- Jeejeebhoy Government Medical College- Johns Hopkins University Clinical Research Site, Pune, India; 4 Johns Hopkins University–India office, Pune, India; 5 Department of Medicine, Byramjee Jeejeebhoy Government Medical College, Pune, India; 6 Johns Hopkins Bloomberg School of Public Health, Baltimore, United States of America; 7 Department of Chest Medicine, Government Headquarters Hospital, Thiruvallur, India; 8 Indian Council of Medical Research, New Delhi, India; Chinese Academy of Medical Sciences and Peking Union Medical College, CHINA

## Abstract

**Background:**

World Health Organization (WHO) recommends systematic screening of high-risk populations, including household contacts (HHCs) of adult pulmonary tuberculosis (TB) patients, as a key strategy for elimination of TB. QuantiFERON-TB Gold In-Tube (QFT-GIT) assay and tuberculin skin test (TST) are two commonly used tools for the detection of latent tuberculosis infection (LTBI) but may yield differential results, affecting eligibility for TB preventive therapy.

**Materials and methods:**

A prospective cohort study of adult pulmonary TB patients and their HHCs were recruited in 2 cities of India, Pune and Chennai. HHCs underwent QFT-GIT (QIAGEN) and TST (PPD SPAN 2TU/5TU). A positive QFT-GIT was defined as value ≥0.35 IU/ml and a positive TST as an induration of ≥5 mm. A secondary outcome of TST induration ≥10mm was explored. Proportion positive by either or both assays, discordant positives and negatives were calculated; test concordance was assessed using percentage agreement and kappa statistics; and risk factors for concordance and discordance including age categories were assessed using logistic regression. Sensitivity and specificity was estimated by latent class model.

**Results:**

Of 1048 HHCs enrolled, 869 [median (IQR) age: 27 years (15–40)] had both TST and QFT-GIT results available and prevalence of LTBI by QFT-GIT was 54% [95% CI (51, 57)], by TST was 55% [95% CI (52, 58)], by either test was 74% [95% CI (71, 77) and by both tests was 35% [95% CI (31, 38)]. Discordance of TST+/QFT-GIT- was 21% while TST-/QFT-GIT+ was 26%. Poor to fair agreement occurred with TST 5mm or 10mm cutoff (60 and 61% agreement with kappa value of 0.20 and 0.25 respectively). Test agreement varied by age, TST strength and induration cut-off. In multivariate analysis, span PPD was a risk factor for QFT-GIT+ and TST- while absence of BCG scar was for TST+ and QFT-GIT-. Being employed and exposure to TB case outside the household case were associated with positivity by both the tests. Sensitivity of TST and QFT-GIT to diagnose LTBI was 77% and 69%. Probability of having LTBI was >90% when both tests were positive irrespective of exposure gradient.

**Conclusion:**

Prevalence of LTBI among HHCs of adult pulmonary TB patients in India is very high and varies by test type, age, and exposure gradient. In our high TB burden setting, a strategy to treat all HHCs or a targeted strategy whereby an exposure index is used should be assessed in future preventive therapy and vaccine studies as HHCs have several factors that place them at high risk for progression to TB disease.

## Introduction

Latent Tuberculosis infection (LTBI) is defined as a state of persistent immune response to stimulation by *Mycobacterium tuberculosis (M*.*tb)* antigens without evidence of clinically manifested active tuberculosis (TB) [1]. The vast majorities of infected persons have no signs or symptoms of TB disease and are not infectious, but they are at risk for developing active TB and becoming infectious. Hence, screening and treatment of LTBI should be an important part of global TB control activities if we want to achieve End TB strategy [2]. The World Health Organization (WHO) recommends systematic screening, identification and treatment of LTBI especially in groups at high risk for developing active TB like people living with HIV, child contacts of pulmonary TB cases, patients with silicosis and other forms of immunosuppression [3]. After ruling out active TB by a symptom screen, individuals should be tested for LTBI by either interferon-gamma release assays (IGRA) or tuberculin skin test (TST).

Both TST and IGRA, the two currently available tests for diagnosis of LTBI, work on the principle of cell-mediated immunity [4]. The TST detects *M*.*tb* sensitization via a delayed-type hypersensitivity response to *M*.*tb* antigens from purified protein derivatives while IGRAs measure interferon-gamma (IFN-γ) release in response to specific *M*.*tb* antigens [4]. Where antigens used in TST may cross-react with environmental non-tuberculous mycobacteria and the BCG vaccine, IGRA claims to overcome these limitations and be more specific than TST. However, concerns have been raised about the accuracy of IGRA and a higher rate of indeterminate results [5, 6].

WHO recommends that either TST or IGRA can be used to test for LTBI in high-income and upper middle-income countries with estimated TB incidence of less than 100 per 100,000. It also suggests that IGRA should not replace TST in low-income and other middle-income countries as the quality of evidence for the recommendation is low [3]. There is limited head-to-head comparison of the two tests especially in low- and middle-income countries with high burden of TB making it unclear which test identifies LTBI better [7]. The true accuracy of these tests can only be assessed by estimating their ability to predict development of active TB, requiring a longer duration of follow-up, which may not be feasible in resource limited settings. The present study was planned to evaluate the diagnostic performance of one type of commercially available IGRA, the QuantiFERON Gold In-Tube assay (QFT-GIT) and TST among the household contacts (HHC) of pulmonary TB patients in a TB endemic setting and determine the factors associated with agreement between these two tests.

## Materials and methods

### Study design and setting

This study was conducted as part of an ongoing prospective study ‘CTRIUMPh’, the details of which are described elsewhere [8]. In brief, CTRIUMPh is a prospective cohort study of adult pulmonary TB cases and their HHCs to evaluate the response to anti-TB treatment in the active TB Cohort and to evaluate *M*.*tb* infection and progression to TB disease in the HHC cohort. CTRIUMPh is enrolling at the National Institute for Research in Tuberculosis, Chennai and Byramjee Jeejeebhoy Government Medical College, Pune, India since August 2014, through academic and operational partnerships with the Johns Hopkins University (JHU), USA.

### Patient population

HHCs of adult pulmonary TB patients, in whom active TB was ruled out, were enrolled and evaluated in the study. We defined HHCs as adults and children living in the same household as the index case during the 3 months prior to diagnosis of the index TB case. HHC who refused to have blood drawn or TST tested were excluded from this analysis.

### Study procedure

All participants provided informed written consent and demographic details, completed a TB risk assessment questionnaire and underwent a physical examination. Sputum samples for smear and culture of acid-fast TB bacilli, blood sample for QFT-GIT, TST and chest x-ray were done for all participants at study entry.

### QFT-GIT

Blood was collected for QFT-GIT assay on the same day when TST was performed but prior to placement of TST. Venous blood samples were collected and processed according to the manufacturer’s instructions (QIAGEN, Germany) by trained laboratory staff. IFN-ɣ levels (IU/ml) were estimated using an ELISA reader (ELx808, BioTek, USA). The results were analyzed using QFT-GIT analysis software (Version: 2.62) and considered positive if the value of the TB Antigen minus Nil control was ≥0.35 IU/ml and ≥25% of nil value.

### TST

The TST was performed by trained staff according to the Mantoux technique [9]. On enrollment to the study, 0.1ml of 2TU / 5TU of PPD {RT23; Statens Serum Institute (SSI), Copenhagen, Denmark or Span diagnostic, India (due to non-availability of the same PPD)} was injected intra dermally on the volar aspect of the forearm. After 48–72 hours, the transverse diameter of the TST induration was read by two trained staff [9]. The result was considered positive if the induration diameter was ≥ 5mm [10]. Secondary outcome of TST induration of ≥10mm was also explored [11].

### TB Risk assessment score

Well-quantified TB exposure is a good surrogate measure of *M*. *tb* infection in HHC in a high-burden setting [12]. A TB risk assessment score was derived using the exposure factors of the adult to the index case. This included a set of 10 questions namely, is the index case spouse of the adult HHC? Is the adult the index case’s primary caregiver? Does the index case sleep in same room / same bed as the adult? Does the index case cough? Have they been reported as PTB? Is the index case smear positive TB? Does the adult live in the same household as the index case? Do they see the index case every day? Are there any other adult TB patients in the household? For HHCs aged 15 years or below, most of these questions were used to assess their exposure with slight modifications in few questions like—is the index case the child’s mother or father? Is the index case the child’s primary caregiver? Does the index case sleep in same room / same bed as the child? Does the child live in the same household as the index case? Does the child see the index case every day? HHCs who had a TB Exposure score greater than 50^th^ percentile were considered as highly exposed and others as low exposure.

### Latent TB infection

LTBI was defined as the presence of a positive TST or QFT-GIT test result at baseline or study entry without evidence of clinical or radiological evidence of TB disease. Prevalence of LTBI was calculated as number of positives by either TST or QFT-GIT tests divided by total number of HHC tested.

### Data analysis

Comparison between groups and concordance between TST and QFT-GIT was performed using Fisher’s exact test. Kappa statistics were used to test the agreement between TST and QFT-GIT [13]. Factors associated with concordance and discordance between the two tests (TST and QFT-GIT) were measured using logistic regression by enter method. All the variables that were significant in the univariate analysis and those that were clinically meaningful were included in the multiple logistic regression. Odds ratio were estimated and adjusted for clustering at the household level. To estimate the sensitivity and specificity of these assessments, in the absence of a gold standard, Latent class analysis was performed for the two diagnostic tests along with the level of exposure. Parameters of interest i.e., true- and false- positive rates of the tests as well as the prevalence of the disease were estimated by modelling the relationship between an unobservable (latent) and observable variable. From these measures, we estimated a ‘consensus’ gold standard i.e., latent class to evaluate sensitivity and specificity of the tests as well as the prevalence of the disease [14]. The program “random LCA” package for R was used to fit latent class analysis model and STATA 15.0 (StataCorp, College Station, Texas, USA) was used for statistical analysis [15]. A p value of <0.05 was considered significant.

The study was approved by the Institutional Review board at both National Institute for Research in Tuberculosis, Chennai and Byramjee Jeejeebhoy Government Medical College, Pune, India and written informed consent was obtained from all participants before study enrollment.

## Results

**Fig 1:**
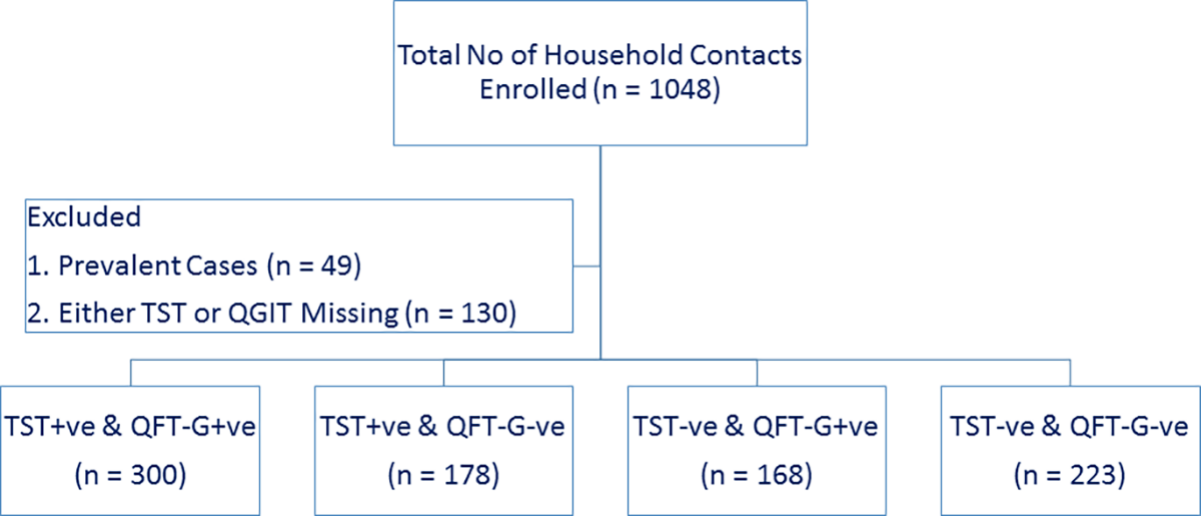
Flow diagram of the study population.

A total of 1048 HHCs [median age: 27 years (IQR: 15–40 years); 55% males and 58% employed] of adult pulmonary TB patients diagnosed with TB and started on anti-TB treatment, were recruited in the HHCs cohort of CTRIUMPh study. Of them, 49 HHCs were diagnosed with active TB while screening for the study while 130 HHCs had either a TST or QFT-GIT test result unavailable. Hence, 869 (83%) HHCs were considered for further analysis (Fig 1: Flow diagram of Study population). PPD RT23 from Statens Serum Institute, Copenhagen, Denmark was used in 124 HHC (14%) in the early part of the study. This PPD was used only for 10months. Subsequently, due to the non-availability of this PPD, we had to switch to locally available Span diagnostic PPD for the skin testing and it was used in the remaining 745 HHCs (86%) in the study.

### Prevalence of LTBI

Prevalence of LTBI, as defined by either a positive TST (induration ≥ 5mm) or positive QFT-GIT (≥0.35 IU/L), was 74% (646/869). At baseline, 55% (478/869) of HHC had a positive TST, 54% (468/869) had a positive QFT-GIT while 35% (300/869) were positive by both tests. **Table 1** shows the factors associated with a positive TST or QFT-GIT assay. Increasing age, body mass index, presence of an employment, diabetes, smoking habit and an absence of BCG scar were associated with higher rates of TST or QFT-GIT positivity. HHC of culture positive pulmonary TB patients had a higher rate of TST positivity.

**Table 1:** Factors associated with positive TST and /or QFT-GIT assays.

Variables	TST			QFT-GIT		
	Neg. (n = 391)	Pos. (n = 478)	Sig.	Neg. (n = 401)	Pos. (n = 468)	Sig.
Age (years)						
<6 years	38 (61.3%)	24 (38.7%)	<0.001	44 (71.0%)	18 (29.0%)	<0.001
6–15 years	94 (57.3%)	70 (42.7%)		99 (60.4%)	65 (39.6%)	
15–45	216 (41.9%)	299 (58.1%)		216 (41.9%)	299 (58.1%)	
>45	43 (33.6%)	85 (66.4%)		42 (32.8%)	86 (67.2%)	
Gender						
Male	217 (45.2%)	263 (54.8%)	>0.950	226 (47.1%)	254 (52.9%)	0.539
Female	174 (44.7%)	215 (55.3%)		175 (45.0%)	214 (55.0%)	
Employed (Housewife, Retired & Student included in No)						
Yes	207 (41.0%)	298 (59.0%)	0.006	207 (41.0%)	298 (59.0%)	<0.001
No	184 (50.5%)	180 (49.5%)		194 (53.3%)	170 (46.7%)	
Body Mass Index (kg/m2)						
<18.5	161 (53.1%)	142 (46.9%)	0.001	167 (55.1%)	136 (44.9%)	<0.001
18.5–24.9	136 (41.5%)	192 (58.5%)		142 (43.3%)	186 (56.7%)	
>24.9	82 (38%)	134 (62%)		83 (38.4%)	133 (61.6%)	
BCG Scar						
Present	235 (48.7%)	248 (51.3%)	0.016	223 (46.2%)	260 (53.8%)	>0.950
Absent	156 (40.4%)	230 (59.6%)		178 (46.1%)	208 (53.9%)	
Smoker						
Current	22 (45.8%)	26 (54.2%)	<0.001	20 (41.7%)	28 (58.3%)	<0.001
Anytime	5 (26.3%)	14 (73.7%)		3 (15.8%)	16 (84.2%)	
Non-smokers	239 (40.5%)	351 (59.5%)		244 (41.4%)	346 (58.6%)	
NA	125 (59.0%)	87 (41.0%)		134 (63.2%)	78 (36.8%)	
Alcohol Intake						
Yes	46 (42.2%)	63 (57.8%)	<0.001	40 (36.7%)	69 (63.3%)	<0.001
No	220 (40.1%)	328 (59.9%)		227 (41.4%)	321 (58.6%)	
NA	125 (59.0%)	87 (41.0%)		134 (63.2%)	78 (36.8%)	
Diabetes (HbA1cEntry > 6.5 or Known DM or Glucose > = 200)						
Yes	22 (37.9%)	36 (62.1%)	<0.001	25 (43.1%)	33 (56.9%)	<0.001
No	244 (40.7%)	355 (59.3%)		242 (40.4%)	357 (59.6%)	
NA	125 (59.0%)	87 (41.0%)		134 (63.2%)	78 (36.8%)	
HIV						
Positive	7 (43.8%)	9 (56.3%)	>0.950	5 (31.3%)	11 (68.8%)	0.313
Negative	384 (45.0%)	469 (55.0%)		396 (46.4%)	457 (53.6%)	
TST Type						
Span	376 (50.5%)	369 (49.5%)	<0.001	349 (46.8%)	396 (53.2%)	NA
SSI	15 (12.1%)	109 (87.9%)		52 (41.9%)	72 (58.1%)	
TB Contact (Outside Household)						
Yes	28 (38.4%)	45 (61.6%)	0.269	30 (41.1%)	43 (58.9%)	0.392
No	363 (45.6%)	433 (54.4%)		371 (46.6%)	425 (53.4%)	
Average Time Spent with Index per Day						
<1 hr	11 (45.8%)	13 (54.2%)	0.667	14 (58.3%)	10 (41.7%)	0.100
1 to 6 hrs	43 (48.3%)	46 (51.7%)		37 (41.6%)	52 (58.4%)	
>6 to < 12 hrs	148 (42.3%)	202 (57.7%)		171 (48.9%)	179 (51.1%)	
>12 but < 18 hrs	129 (48.5%)	137 (51.5%)		107 (40.2%)	159 (59.8%)	
≥18 hrs	59 (42.8%)	79 (57.2%)		71 (51.4%)	67 (48.6%)	
Don't Know	1 (50.0%)	1 (50.0%)		1 (50.0%)	1 (50.0%)	
Sleeping with Index (After Diagnosis)						
Same room, same bed	106 (42.2%)	145 (57.8%)	0.618	100 (39.8%)	151 (60.2%)	0.028
Same room, diff. bed	163 (44.9%)	200 (55.1%)		169 (46.6%)	194 (53.4%)	
Same house	117 (48.1%)	126 (51.9%)		128 (52.7%)	115 (47.3%)	
Others	5 (41.7%)	7 (58.3%)		4 (33.3%)	8 (66.7%)	
Sharing Meals						
None	14 (41.2%)	20 (58.8%)	0.195	14 (41.2%)	20 (58.8%)	0.531
1	105 (45.7%)	125 (54.3%)		100 (43.5%)	130 (56.5%)	
2	215 (43.0%)	285 (57.0%)		233 (46.6%)	267 (53.4%)	
3 or More	57 (54.3%)	48 (45.7%)		54 (51.4%)	51 (48.6%)	
Primary Care provider						
Yes	97 (49.0%)	101 (51.0%)	0.223	107 (54.0%)	91 (46.0%)	0.012
No	294 (43.8%)	377 (56.2%)		294 (43.8%)	377 (56.2%)	
Index Case Details (Baseline, n = 399)						
Cough Duration	n = 869			n = 869		
<2 weeks	22 (37.9%)	36 (62.1%)	0.322	22 (37.9%)	36 (62.1%)	0.638
2–4 weeks	78 (49.4%)	80 (50.6%)		74 (46.8%)	84 (53.2%)	
4–8 weeks	204 (43.3%)	267 (56.7%)		219 (46.5%)	252 (53.5%)	
>8 weeks	87 (47.8%)	95 (52.2%)		86 (47.3%)	96 (52.7%)	
Cavity on CXR	n = 798			n = 798		
Present	179 (46.0%)	210 (54.0%)	0.133	168 (43.2%)	221 (56.8%)	0.102
Absent	166 (40.6%)	243 (59.4%)		201 (49.1%)	208 (50.9%)	
Sputum Grading	n = 869			n = 869		
Negative	168 (47.2%)	188 (52.8%)	0.556	177 (49.7%)	179 (50.3%)	0.181
<2+	138 (43.7%)	178 (56.3%)		141 (44.6%)	175 (55.4%)	
2+ & More	85 (43.1%)	112 (56.9%)		83 (42.1%)	114 (57.9%)	
Smear & Culture	n = 869			n = 869		
C- S-	65 (57.0%)	49 (43.0%)	0.045	64 (56.1%)	50 (43.9%)	0.102
C- S+	3 (50.0%)	3 (50.0%)		2 (33.3%)	4 (66.7%)	
C+ S-	103 (42.6%)	139 (57.4%)		113 (46.7%)	129 (53.3%)	
C+ S+	220 (43.4%)	287 (56.6%)		222 (43.8%)	285 (56.2%)	

NA: not applicable. Questions about smoking and alcohol use were not asked and HbA1c for diabetes status was not done for participants <15 years of age. Employed: housewife, retired persons & student were included in the category ‘No’. HbA1c –Glycosylated Hemoglobin, Known DM–Self reported diabetes mellitus, CXR–Chest Xray, RBS–Random Blood Sugar

### Performance of TST and QFT-GIT

**Fig 2:**
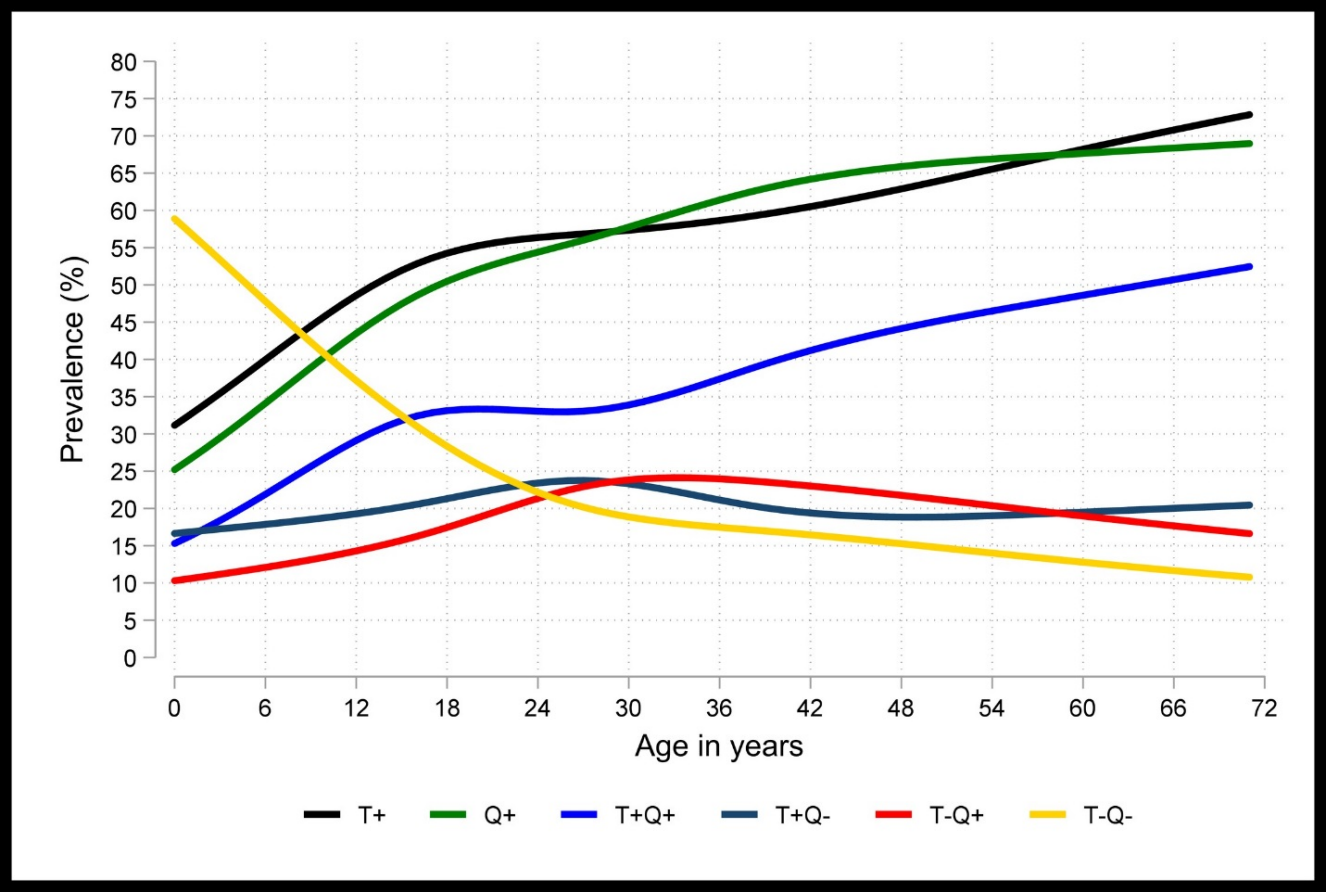
Age-specific prevalence of TST (≥5mm) and QFT-GIT positivity at baseline.

Among the HHCs who had both QFT-GIT and TST results available at baseline, the proportion positive by either of the test was almost similar. Also as age increased a greater proportion of HHCs showed a positive response to these tests (**Fig 2:** Age-specific prevalence of TST and QFT-GIT). The agreement was higher among pediatric age group, with the highest agreement in children below 6 years of age.

### Concordance and discordance between TST and QFT-GIT using different types of TST

50% of HHCs (369/745) who received SPAN Diagnostic PPD and 88% (109/124) of HHCs who received SSI PPD showed positive TST result. With SPAN Diagnostic TST, the concordance with QFT-GIT + was 60% (444/745). The agreement was 60% with a poor kappa of 0.19 (**Table** 2 shows the agreement between TST and QFT-GIT based on type of TST). Using SSI TST, the concordance observed was 64% (79/124); and the agreement was 64% with a kappa of 0.17. (S1 Table). As the number of HHCs who received SSI TST was small, we subsequently combined all the HHCs TST results irrespective of the type of TST received.

**Table 2:** Agreement between TST and QFT-GIT based on the type of TST (TST + ≥ 5mm).

Product	TST	QFT-GIT		Total	Kappa (SE)	
		Pos.	Neg.			
Total	Pos.	300 (34.5%)	178 (20.5%)	478 (55.0%)	60.2%	Poor
	Neg.	168 (19.3%)	223 (25.7%)	391 (45.0%)	0.197 (0.033)	
Span	Pos.	232 (31.1%)	137 (18.4%)	369 (49.5%)	59.6%	Poor
	Neg.	164 (22.0%)	212 (28.5%)	376 (50.5%)	0.192 (0.036)	
SSI	Pos.	68 (54.8%)	41 (33.1%)	109 (87.9%)	63.7%	Poor
	Neg.	4 (3.2%)	11 (8.9%)	15 (12.1%)	0.173 (0.069)	
Total		468 (53.9%)	401 (46.1%)	869 (100%)		

### Concordance between TST and QFT-GIT using various cut-off values for TST induration

Using a TST induration cut-off ≥ 5mm and QFT-GIT ≥ 0.35IU/ml, 55% had a positive TST (478/869) while 54% had a positive QFT-GIT (468/869). The overall agreement between TST ≥5mm and QFT-GIT, was 60% [kappa = 0.2, 95% CI (0.16, 0.23)] **(**S2 Table). Using a higher TST induration cut-off of ≥ 10mm, only 26% (228/869) showed a positive TST. The overall agreement between TST and QFT-GIT was 61% [kappa = 0.25, 95% CI (0.22, 0.28)].

With TST induration cut-off of 5mm, 35% had both TST+/QFT-GIT+ while with the induration cut-off increased to 10mm, only 21% had both TST+/QFT-GIT+. Concordance between TST and QFT-GIT was higher among HHCs < 14 years of age as well as when the TST cut-off for a positive result was fixed at 10mm [TST+/QFT-GIT+: 77%, kappa = 0.44, 95% CI (0.38, 0.51)] **(**S2 Table**)**.

### Discordance between TST and QFT-GIT

Discordance between the two tests was seen among all age groups. TST+/ QFT-GIT- results were observed in 178 (21%) of HHCs (19% in children < 6 years and 19% in adults > 45 years of age) while TST-/QFT-GIT+ results were observed in 168 (19%) of HHC. This was lesser across all age groups as compared to TST+/QFT-GIT-. (**Table 3** shows the age-specific agreement of TST and QFT-GIT assay).

**Table 3:** Age-specific agreement of TST and QFT-GIT assays.

	Age				Total (n = 869)	p Value
	<6 (n = 62)	6–15 (n = 164)	15–45 (n = 515)	>45 (n = 128)		
TST (T+)						
Pos.	24 (39%)	70 (43%)	299 (58%)	85 (66%)	478 (55%)	<0.001
Neg.	38 (61%)	94 (57%)	216 (42%)	43 (34%)	391 (45%)	
QFT-GIT (Q+)						
Pos.	18 (29%)	65 (40%)	299 (58%)	86 (67%)	468 (54%)	<0.001
Neg.	44 (71%)	99 (60%)	216 (42%)	42 (33%)	401 (46%)	
LTBI (T+ / Q+)						
Pos.	30 (48%)	93 (57%)	413 (80%)	110 (86%)	646 (74%)	<0.001
Neg.	32 (52%)	71 (43%)	102 (20%)	18 (14%)	223 (26%)	
Only TST (T+ & Q-)						
Yes	12 (19%)	28 (17%)	114 (22%)	24 (19%)	178 (21%)	0.509
No	50 (81%)	136 (83%)	401 (78%)	104 (81%)	691 (80%)	
Only QFT-GIT (T- & Q+)						
Yes	6 (10%)	23 (14%)	114 (22%)	25 (20%)	168 (19%)	0.026
No	56 (90%)	141 (86%)	401 (78%)	103 (81%)	701 (81%)	
Both (T+ & Q+)						
Yes	12 (19%)	42 (26%)	185 (36%)	61 (48%)	300 (35%)	<0.001
No	50 (81%)	122 (74%)	330 (64%)	67 (52%)	569 (66%)	
Agreement	71%	69%	56%	62%	60%	
Kappa	0.36 (0.12) Fair	0.36 (0.07) Fair	0.09 (0.04) Poor	0.14 (0.09) Poor	0.20 (0.03) Poor	

### Correlates of concordance and discordance between QFT-GIT and TST

Various risk factors that were considered to be associated with TST and QFT-GIT concordance were analyzed. After adjusting for the household level clustering and factors significant in the univariate analysis, being employed [aOR 1.5, 95% CI (1.0, 2.3)], and exposure to a TB case outside the household [aOR 1.6, 95% CI (1.0, 2.6)] were significantly associated with a higher chance of either test discordance (**Table 4** shows factors associated with concordance and discordance of TST and QFT-GIT). Absence of BCG scar was found to be associated with higher chance of being TST+ and QFT-GIT- [aOR 1.5; 95% CI (1.0, 2.2)] (S3 Table). Type of TST product used i.e., SPAN Diagnostic TST versus SSI TST tended towards higher discordance between the tests i.e., TST- and QFT-GIT+ [aOR 7.7, 95%CI (2.2, 27.4)] (S4 Table).

**Table 4:** Factors associated with concordance and discordance between QFT-GIT and TST.

Factors	T+Q+/T-Q-	T+Q-/T-Q+	OR (95% CI)	p Value	aOR (95% CI)	p Value
Age (in years)						
<6 years	44 (8%)	18 (5%)	1.00		1.00	
6–15 years	113 (22%)	51 (15%)	1.1 (0.6–2.1)	0.77	1.0 (0.5–2.1)	0.95
15–45	287 (55%)	228 (66%)	1.9 (1.1–3.6)	0.03	1.0 (0.2–4.3)	0.98
>45	79 (15%)	49 (14%)	1.5 (0.8–3.0)	0.40	0.6 (0.1–3.0)	0.56
Employment						
Yes	279 (53%)	226 (65%)	1.6 (1.3–2.2)	<0.001	1.5 (1.0–2.3)	0.048
No	244 (47%)	120 (35%)	1.00		1.00	
Body Mass Index (kg/m^2^)						
<18.5	203 (40%)	100 (30%)	1.00		1.00	
18.5–24.9	184 (36%)	144 (43%)	1.6 (1.1–2.2)	0.005	1.3 (0.8–1.9)	0.30
>24.9	123 (24%)	93 (28%)	1.5 (1.1–2.2)	0.018	1.1 (0.7–1.8)	0.58
BCG Scar						
Present	305 (58%)	178 (51%)	1.00		1.00	
Absent	218 (42%)	168 (49%)	1.3 (1.0–1.7)	0.049	1.2 (0.9–1.7)	0.22
Type of PPD						
Span	444 (85%)	301 (87%)	1.2 (0.8–1.8)	0.44	1.3 (0.8–2.1)	0.22
SSI	79 (15%)	45 (13%)	1.00		1.00	
Smoker						
Current	24 (5%)	24 (7%)	1.3 (0.7–2.4)	0.34	1.4 (0.6–3.0)	0.39
Anytime	13 (3%)	6 (2%)	0.6 (0.3–1.5)	0.28	0.6 (0.2–1.6)	0.33
Non smokers	337 (64%)	253 (73%)	1.00		1.00	
NA	149 (29%)	63 (18%)	0.6 (0.4–0.8)	0.001	0.9 (0.2–3.1)	0.84
Alcohol use						
Yes	61 (12%)	48 (14%)	1.0 (0.7–1.6)	0.83	0.8 (0.4–1.3)	0.36
No	313 (60%)	235 (68%)	1.00		1.00	
NA*	149 (29%)	63 (18%)	0.6 (0.4–0.8)	0.001		
TB Contact (Outside Household)						
Yes	35 (7%)	38 (11%)	1.7 (1.1–2.7)	0.018	1.6 (1.0–2.6)	0.046
No	488 (93%)	308 (89%)	1.00		1.00	
Sleeping with Index						
Same room & bed	157 (30%)	94 (27%)	1.00		1.00	
Same room, diff. bed	217 (42%)	146 (42%)	1.1 (0.8–1.6)	0.49	1.3 (0.9–1.8)	0.20
Same house, diff. room	144 (28%)	99 (29%)	1.1 (0.8–1.6)	0.45	1.3 (0.9–2.0)	0.16
Others	5 (1%)	7 (2%)	2.3 (0.7–8.0)	0.18	2.9 (0.8–11.0)	0.12
Index Cavity on CXR						
Present	238 (49%)	151 (48%)	0.9 (0.7–1.3)	0.69	0.9 (0.7–1.3)	0.64
Absent	244 (51%)	165 (52%)	1.00		1.00	
Smear & Culture						
C- S-	75 (14%)	39 (11%)	1.00		1.00	
C- S+	5 (1%)	1 (0.3%)	0.4 (0.1–1.8)	0.22	0.3 (0.1–1.4)	0.13
C+ S-	142 (27%)	100 (29%)	1.4 (0.8–2.3)	0.25	1.5 (0.8–2.7)	0.18
C+ S+	301 (58%)	206 (60%)	1.3 (0.8–2.2)	0.28	1.4 (0.8–2.4)	0.26

*Not applicable in Alcoholic omitted because of collinearity. Odds ratios were adjusted for Household cluster

### Effect of BCG vaccination on TST and QFT-GIT performance

BCG vaccination of the HHCs was ascertained by the presence or absence of BCG scar in their left deltoid, by trained readers. Presence of scar in the deltoid of a HHCs was considered to be BCG vaccinated. We found that performance of QFT-GIT was not affected by presence or absence of BCG vaccination. However, TST induration showed a difference with different cut-offs–with cut-off value of > 5mm, 60% of HHCs with a TST ≥ 5mm did not have a visible BCG scar. **(**S5 Table).

### Estimation of accuracy of QFT-GIT, TST and exposure gradient

Prevalence of LTBI in HHC was estimated by the latent class analysis model and was found to be 60% (95% CI: 42, 78). The predicted frequencies for the patterns of response to the three assessments showed a good fit with the observed data. In the latent class analysis, TST (T) had the highest estimated sensitivity and specificity than the other two assessments (**Table 5** shows sensitivity and specificity of LTBI by Latent Class analysis model). The Exposure score (E) and QFT-GIT (Q), both had a sensitivity close to that of TST, while the estimated specificity of QFT-GIT was closer to TST than the exposure score. When any two tests were compared against the third test, to evaluate differences in their diagnostic accuracy, statistically significant differences were observed for the comparison between the three assessments (p<0.001). The probability of being LTBI was high (94%) when all three assessments were positive as compared to any two or one: T+Q+E- (90%), T+Q-E+ (74%), T+Q-E- (65%), T-Q+E+ (56%), T-Q+E- (45%), T-Q-E+ (20%) and T-Q-E- (14%). Also, by using the latent class analysis model, the probability of being LTBI was higher when TST induration cut-off was used as >10mm than an induration cut-off of 5mm when both QFT-GIT and TST were positive. **(**S6 and S7 Tables).

**Table 5:** Sensitivity and specificity of LTBI by latent class analysis method.

Test	Sensitivity (95% CI)	Specificity (95% CI)
TST (T)*	76%	77%
	(56%–97%)	(57%–98%)
QFT-GIT (Q)	69%	69%
	(55%–83%)	(48%–91%)
Exposure (E)	72%	38%
	(67%–76%)	(32%–44%)

Estimated Prevalence of LTBI = 60% (95%CI: 42%–78%). *Probability of being LTBI Positive*: *T+Q+E+ (94%)*, *T+Q+E- (90%)*, *T+Q-E+ (74%)*, *T+Q-E- (65%)*, *T-Q+E+ (56%)*, *T-Q+E- (45%)*, *T-Q-E+ (20%) and T-Q-E- (14%)*.

*TST induration cut-off used is >5mm as the cut-off. (Analysis using TST cut-off>10mm is given as S6 Table)

## Discussion

Our study recruited a large cohort of HHC of adult pulmonary TB patients to determine the prevalence of LTBI in this high-risk group for TB and evaluated the performance of QFT-GIT and TST to diagnose LTBI. We observed a high prevalence of LTBI among these HHCs, similar to earlier reports [7, 9–11]. Also WHO estimated that approximately 40% of the Indians are infected with *M*.*tb*, the vast majority of who have dormant infection (12).Using TST alone to diagnose LTBI, we estimated the prevalence of LTBI to be 55% while using positivity by either of the two tests, we showed a LTBI prevalence of 72%.

However, the type of TST product used and the cutoff fixed for a positive result impacted the outcome substantially. We noted some differences in the proportion of positives in our exploratory comparison of two different purified protein derivative products used for TST. SSI PPD had a higher positivity rate as well better concordance with QFT-GIT results than SPAN diagnostics. Similarly, by increasing the TST induration cut-off for positivity from 5mm to 10mm, (which is often used as a cutoff for TST positivity in many high TB-burden settings including India) the proportion of individuals with TST positivity reduced significantly to 27%. Also the results vary with different strengths of PPD used for TST. Studies from India have shown that cutaneous hypersensitivity to 1TU or 2TU PPD or 5TU PPD are not comparable [16, 17]. However a study from Philippines on TST reactivity among children with TB, found 2 TU PPD RT-23 to be comparable with 5 TU PPD-S and that one can be used instead of the other in routine Mantoux testing [18].

The study also identified predictors for LTBI. Age of the HHC, employment status, absence of BCG vaccination or exposure to TB bacilli outside the household emerged as important risk factors for the presence of LTBI. Age was a strong correlate for both TST and QFT-GIT positivity than either of the tests alone, which is in contrast to a study performed in Korea [19]. This could be due to the increasing mobility of HHC for work and livelihood thus increasing their exposure to both tuberculous and non-tuberculous mycobacteria in the environment or more recent exposure to TB as has been suggested in some studies [18]. In addition, a higher proportion of individuals with malnutrition or low body mass index, and absence of BCG vaccination scar showed positivity to TST, irrespective of the cut-off, similar to other high risk cohorts [20, 21]. These could also be false positive TST due to environmental or non-tuberculous mycobacteria, commonly encountered with manual laborers or farmers, who form the majority of our population. Poor socioeconomic status and living conditions have also been shown as strong risk factors linked with LTBI [21].

We noted substantial discordance between TST and QFT-GIT with older age groups having poor to fair agreement between the tests. A fair agreement of 61% and a kappa of 0.21 were reported between the two tests in our study population, in par with a recent meta-analysis [22]. Concordance between the two tests was better with a higher cut-off for TST positivity. About a quarter of our HHCs showed discordance between TST and QFT-GIT in our high TB-burden setting and hence it is difficult to say which test works better than the other. Employment status and exposure to TB outside the household index case were most associated with TST/QFT-GIT positive and negative concordance among the risk factors explored. Though QFT-GIT is particularly designed to overcome most of the limitation of TST and is regarded more specific than TST, we were unable to obtain a perfect agreement between the two tests, unlike other studies that have shown a high agreement between the two tests [23]. The main reasons for this could be a higher proportion of HHC with BCG vaccination and higher prevalence of non-tuberculous mycobacteria in our environment that is very different from other population [24].

**Fig 3:**
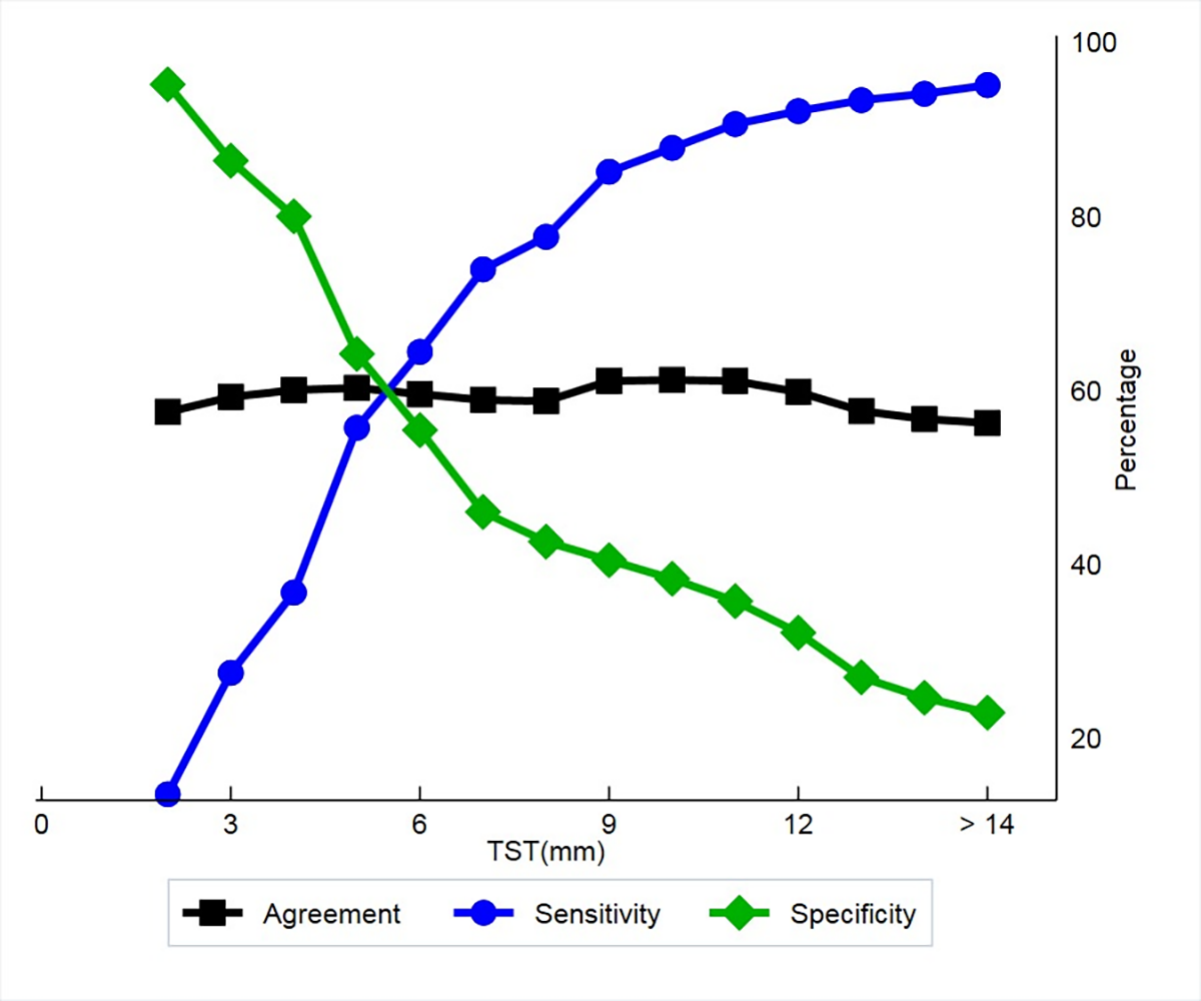
Agreement between TST and QFT-GIT at various induration cut-off.

We also used latent class model to estimate and compare the sensitivity and specificity of TST and QFT-GIT in the absence of a gold standard test. The sensitivity in previously reported studies ranged from 55% to 93% for QFT-GIT with a pooled estimate of 78% [25]. Our estimates of the sensitivity of TST and QFT-GIT for LTBI were around 76% and 69% while specificity was 77% and 69% respectively. Though the sensitivity is almost similar to that observed in a meta-analysis, the specificity is lower than other studies [26, 27]. Unlike these reports, a recently published systematic review using latent class modelling to assess the utility of TST and QFT-GIT for LTBI diagnosis in different population groups showed a low sensitivity (52%) but high specificity (97%) of QFT-GIT to diagnose LTBI [28]. Even active TB studies have reported low sensitivity but varying specificity for QFT-GIT [29, 30]. Furthermore, as the strength of TST product is increased, the induration cut-off for a positive TST gets reduced and is closer to positivity of QFT-GIT. In our cohort, irrespective of the strength of TST, maximum sensitivity and specificity of TST against QFT- GIT was obtained at an induration of >11mm (Fig 3).

Our study showed that the initial results of QFT-GIT are in fair agreement with TST in a TB endemic country. With the use of varying strengths of TST and different induration as cut-off for a positive TST, we will need a better test than TST to diagnose LTBI. This cohort is under follow-up. It needs to be seen how many concordant TST+/QFT-GIT+ individuals actually develop TB disease in the follow-up period. The significance of discordant result with the progression of infection also needs further evaluation.

Few limitations of our study include non-availability of the same strength and manufacturer of TST throughout the study period. Clearly while the individual prevalence by either tests show similar positivity, the LTBI tests are not identifying the same populations as being LTBI positive. It remains to be seen with longitudinal follow-up what the significance and value is of using both tests in combination. Lastly, an inherent limitation of QFT-GIT is its inability to differentiate between recent or remote infection.

### Implication of the study

With countries pursuing the goal of TB elimination, this article highlights the burden and the need to address LTBI if further progress has to be made in eliminating the disease, beyond newer drugs and regimens. The article highlights an urgent need to identify better diagnostics for the diagnosis of LTBI.

## Conclusions

In conclusion, our study showed a high prevalence of LTBI among HHC of pulmonary TB patients in a TB high burden setting. By performing two LTBI tests, detailed exposure assessments and characterization of the HHCs, we add a more nuanced understanding of LTBI and correlates of LTBI in India, the country with the world’s largest burden of TB and LTBI. The two diagnostic tests currently available to diagnose LTBI have only poor to fair concordance and vary with age-group of the population. With the lack of a gold standard and due to varying sensitivity and specificity of the currently available tests, the value of using both tests in combination needs further study particularly in TB endemic countries like India, that are scaling-up TB preventive therapy under programme settings.

## Supporting information

S1 Table Agreement between TST and QFT-GIT based on the type of TST and TST + ≥10mm. (DOCX)

S2 Table Agreement between TST and QFT-GIT based on the induration cut-off. (DOCX)

S3 Table Factors associated with discordance between TST and QFT-GIT (TST positive & QFT-GIT negative). (DOCX)

S4 Table Factors associated with discordance between QFT-GIT and TST (TST negative & QFT-GIT positive). (DOCX)

S5 Table Table showing response to TST and QFT-GIT among those who had BCG vaccination. (DOCX)

S6 Table Sensitivity and specificity of LTBI by latent class analysis method (with TST >10mm). (DOCX)

S7 Table Consolidated table of data pattern and probability of LTBI by latent class analysis method. (DOCX)
