# Supplementary material for: Tuberculin skin test and QuantiFERON-Gold In Tube assay for diagnosis of latent TB infection among household contacts of pulmonary TB patients in high TB burden setting
Source: PLoS One. 2018 Aug 1;13(8):e0199360. doi: 10.1371/journal.pone.0199360 (PMC6070176; doi:10.1371/journal.pone.0199360)
Supplement: S1 Table — (DOCX) [file pone.0199360.s001.docx]

**S1 Table: Agreement between TST and QFT-GIT based on the Type of TST (TST+ ≥ 10mm)**

| Product | TST | QFT-GIT | | Total | Agreement & Kappa (SE) | |
| --- | --- | --- | --- | --- | --- | --- |
|  |  | Pos. | Neg. |  |  |  |
| Total | Pos. | 300 (34.5%) | 178 (20.5%) | 478 (55.0%) | 60.2% | Poor |
|  | Neg. | 168 (19.3%) | 223 (25.7%) | 391 (45.0%) | 0.197 (0.033) | |
| Span | Pos. | 232 (31.1%) | 137 (18.4%) | 369 (49.5%) | 59.6% | Poor |
|  | Neg. | 164 (22.0%) | 212 (28.5%) | 376 (50.5%) | 0.192 (0.036) | |
| SSI | Pos. | 68 (54.8%) | 41 (33.1%) | 109 (87.9%) | 63.7% | Poor |
|  | Neg. | 4 (3.2%) | 11 (8.9%) | 15 (12.1%) | 0.173 (0.069) | |
| Total | | 468 (53.9%) | 401 (46.1%) | 869 (100%) |  |  |
