# Supplementary material for: Tuberculin skin test and QuantiFERON-Gold In Tube assay for diagnosis of latent TB infection among household contacts of pulmonary TB patients in high TB burden setting
Source: PLoS One. 2018 Aug 1;13(8):e0199360. doi: 10.1371/journal.pone.0199360 (PMC6070176; doi:10.1371/journal.pone.0199360)
Supplement: S2 Table — (DOCX) [file pone.0199360.s002.docx]

**S2 Table: Agreement between TST and QFT-GIT based on the Induration cut-off**

| Test+_ve_ | TST ≥5mm or QFT-GIT Positive | | | | TST ≥10mm or QFT-GIT Positive | | | |
| --- | --- | --- | --- | --- | --- | --- | --- | --- |
|  | Over all (n=869) | | <14 Years (n=196) | | Over all (n=869) | | <14 Years (n=196) | |
|  | Pos. | %(95% CI) | Pos. | %(95% CI) | Pos. | %(95% CI) | Pos. | %(95% CI) |
| LTBI | 646 | 74(71 - 77) | 104 | 53(46 – 60) | 517 | 60(56 - 63) | 76 | 39(32 - 46) |
| TST Alone | 178 | 21(18 - 23) | 33 | 17(12 - 22) | 49 | 6(4 - 7) | 5 | 3(0.3 - 5) |
| TST | 478 | 55(52 - 58) | 77 | 39(32 - 46) | 228 | 26(23 - 29) | 36 | 18(13 - 24) |
| QFT-GIT Alone | 168 | 19(17 – 22) | 27 | 14(9 - 19) | 168 | 19(17 - 22) | 27 | 14(9 - 19) |
| QFT-GIT | 468 | 54(51 - 57) | 71 | 36(30 – 43) | 468 | 54(51 - 57) | 71 | 36(30 – 43) |
| Both | 300 | 35(31 - 38) | 44 | 22(17 - 28) | 179 | 21(18 - 23) | 31 | 16(11 - 21) |
| Agree* | 60% | Poor | 69% | Fair | 61% | Fair | 77% | Moderate |
| Kappa (SE) | 0.197 (0.03) | | 0.350 (0.07) | | 0.250 (0.03) | | 0.440 (0.06) | |

*Agreement between TST & QFT-GIT; Confidence interval was based on Binomial Exact.
