# Supplementary material for: Tuberculin skin test and QuantiFERON-Gold In Tube assay for diagnosis of latent TB infection among household contacts of pulmonary TB patients in high TB burden setting
Source: PLoS One. 2018 Aug 1;13(8):e0199360. doi: 10.1371/journal.pone.0199360 (PMC6070176; doi:10.1371/journal.pone.0199360)
Supplement: S3 Table — (DOCX) [file pone.0199360.s003.docx]

**S3 Table: Factors associated with Discordance between TST and QFT-GIT (TST positive & QFT-GIT negative)**

| Factors | T+Q+/T-Q- | T+Q- | OR(95% CI) | p Value | aOR(95% CI) | p Value |
| --- | --- | --- | --- | --- | --- | --- |
| Age (years) | | | | | | |
| <6 years | 44 (8%) | 12 (7%) | 1.00 |  | 1.00 |  |
| 6 – 15 years | 113 (22%) | 28 (16%) | 0.9 (0.4 - 2.0) | 0.81 | 0.9 (0.4 - 2.0) | 0.74 |
| 15 - 45 | 287 (55%) | 114 (64%) | 1.5 (0.7 - 3.0) | 0.30 | 0.8 (0.2 - 4.7) | 0.85 |
| >45 | 79 (15%) | 24 (14%) | 1.1 (0.5 - 2.6) | 0.80 | 0.6 (0.1 - 3.4) | 0.55 |
| Employment | | | | | | |
| Yes | 279 (53%) | 113 (64%) | 1.5 (1.1 - 2.2) | 0.03 | 1.5 (0.9 - 2.6) | 0.13 |
| No | 244 (47%) | 65 (37%) | 1.00 |  | 1.00 |  |
| Body Mass Index (kg/m^2^) | | | | | | |
| <18.5 | 203 (40%) | 53 (30%) | 1.00 |  | 1.00 |  |
| 18.5 - 24.9 | 184 (36%) | 75 (43%) | 1.6 (1.0 - 2.4) | 0.04 | 1.3 (0.8 - 2.2) | 0.36 |
| >24.9 | 123 (24%) | 47 (27%) | 1.5 (0.9 - 2.3) | 0.11 | 1.1 (0.6 - 2.0) | 0.82 |
| BCG Scar | | | | | | |
| Present | 305 (58%) | 83 (47%) | 1.00 |  | 1.00 |  |
| Absent | 218 (42%) | 95 (53%) | 1.6 (1.1 - 2.3) | 0.009 | 1.5 (1.0 - 2.2) | 0.04 |
| PPD | | | | | | |
| Span | 444 (85%) | 137 (77%) | 0.6 (0.4 - 0.9) | 0.03 | 0.7 (0.4 - 1.1) | 0.15 |
| SSI | 79 (15%) | 41 (23%) | 1.00 |  | 1.00 |  |
| Smoker | | | | | | |
| Current | 24 (5%) | 11 (6%) | 1.2 (0.6 - 2.6) | 0.64 | 1.3 (0.5 - 3.3) | 0.54 |
| Anytime | 13 (3%) | 2 (1%) | 0.4 (0.1 - 1.6) | 0.28 | 0.6 (0.1 - 2.3) | 0.42 |
| Non-smokers | 337 (64%) | 129 (73%) | 1.00 |  | 1.00 |  |
| NA | 149 (29%) | 36 (20%) | 0.6 (0.4 - 1.0) | 0.04 | 1.0 (0.2 - 4.3) | 0.98 |
| Alcoholic | | | | | | |
| Yes | 61 (12%) | 21 (12%) | 0.9 (0.5 - 1.6) | 0.69 | 0.7 (0.4 - 1.4) | 0.33 |
| No | 313 (60%) | 121 (68%) | 1.00 |  | 1.00 |  |
| NA* | 149 (29%) | 36 (20%) | 0.6 (0.4 - 1.0) | 0.05 |  | |
| TB Contact (Outside Household) | | | | | | |
| Yes | 35 (7%) | 20 (11%) | 1.8 (1.0 - 3.3) | 0.07 | 1.6 (0.8 - 3.1) | 0.14 |
| No | 488 (9%) | 158 (89%) | 1.00 |  | 1.00 |  |
| Sleeping with Index (After Diagnosis) | | | | | | |
| Same room, same bed | 157 (30%) | 44 (25%) | 1.00 |  | 1.00 |  |
| Same room, diff. bed | 217 (42%) | 76 (43%) | 1.2 (0.8 - 1.9) | 0.28 | 1.5 (0.9 - 2.3) | 0.10 |
| Same house, diff. room | 144 (28%) | 55 (31%) | 1.4 (0.9 - 2.2) | 0.19 | 1.5 (0.9 - 2.5) | 0.09 |
| Others | 5 (1%) | 3 (2%) | 2.1 (0.4 - 11.9) | 0.38 | 3.0 (0.6 - 15.9) | 0.19 |
| INDEX Cavity on CXR | | | | | | |
| Present | 238 (49%) | 70 (41%) | 0.7 (0.5 - 1.1) | 0.10 | 0.7 (0.5 - 1.1) | 0.12 |
| Absent | 244 (51%) | 100 (59%) | 1.00 |  | 1.00 |  |
| Smear & Culture | | | | | | |
| C- S- | 75 (14%) | 19 (11%) | 1.00 |  | 1.00 |  |
| C- S+ | 5 (1%) | 0 (0%) | NA | | NA | |
| C+ S- | 142 (27%) | 55 (31%) | 1.5 (0.7 - 3.3) | 0.27 | 1.3 (0.6 - 2.9) | 0.49 |
| C+ S+ | 301 (58%) | 104 (58%) | 1.4 (0.7 - 2.8) | 0.40 | 1.3 (0.6 - 2.8) | 0.45 |

^*^Not applicable in Alcoholic omitted because of collinearity. Odds ratios were adjusted for Household cluster.
