# Supplementary material for: Tuberculin skin test and QuantiFERON-Gold In Tube assay for diagnosis of latent TB infection among household contacts of pulmonary TB patients in high TB burden setting
Source: PLoS One. 2018 Aug 1;13(8):e0199360. doi: 10.1371/journal.pone.0199360 (PMC6070176; doi:10.1371/journal.pone.0199360)
Supplement: S4 Table — (DOCX) [file pone.0199360.s004.docx]

**S4 Table: Factors associated with Discordance between QFT-GIT and TST (TST negative & QFT-GIT positive)**

| Factors | T+Q+/T-Q- | T-Q+ | OR(95% CI) | p Value | aOR(95% CI) | p Value |
| --- | --- | --- | --- | --- | --- | --- |
| Age (years) | | | | | | |
| <6 years | 44 (8%) | 6 (4%) | 1.00 |  | 1.00 |  |
| 6 – 15 years | 113 (22%) | 23 (14%) | 1.5 (0.6 – 4.0) | 0.42 | 1.2 (0.4 - 3.6) | 0.68 |
| 15 - 45 | 287 (55%) | 114 (68%) | 2.9 (1.2 - 7.2) | 0.02 | 1.3 (0.2 - 9.4) | 0.80 |
| >45 | 79 (15%) | 25 (15%) | 2.3 (0.8 - 6.4) | 0.10 | 0.8 (0.1 - 6.2) | 0.84 |
| Employment | | | | | | |
| Yes | 279 (53%) | 113 (67%) | 1.8 (1.3 - 2.6) | 0.001 | 1.4 (0.8 - 2.5) | 0.25 |
| No | 244 (47%) | 55 (33%) | 1.00 |  | 1.00 |  |
| Body Mass Index (kg/m^2^) | | | | | | |
| <18.5 | 203 (40%) | 47 (29%) | 1.00 |  | 1.00 |  |
| 18.5 - 24.9 | 184 (36%) | 69 (43%) | 1.6 (1.1 - 2.4) | 0.02 | 1.2 (0.7 - 2.1) | 0.49 |
| >24.9 | 123 (24%) | 46 (28%) | 1.6 (1.0 - 2.5) | 0.03 | 1.2 (0.7 - 2.1) | 0.49 |
| BCG Scar | | | | | | |
| Present | 305 (58%) | 95 (57%) | 1.00 |  | 1.00 |  |
| Absent | 218 (42%) | 73 (44%) | 1.1 (0.8 - 1.5) | 0.69 | 0.9 (0.6 - 1.4) | 0.76 |
| PPD | | | | | | |
| Span | 444 (85%) | 164 (98%) | 7.3 (2.2 - 24.7) | 0.001 | 7.7 (2.2 - 27.4) | 0.002 |
| SSI | 79 (15%) | 4 (2%) | 1.00 |  | 1.00 |  |
| Smoker | | | | | | |
| Current | 24 (5%) | 13 (8%) | 1.5 (0.7 - 2.9) | 0.27 | 1.5 (0.5 - 3.9) | 0.45 |
| Anytime | 13 (3%) | 4 (2%) | 0.8 (0.3 - 2.5) | 0.75 | 0.7 (0.2 - 2.3) | 0.57 |
| Non smokers | 337 (64%) | 124 (74%) | 1.00 |  | 1.00 |  |
| NA | 149 (29%) | 27 (16%) | 0.5 (0.3 - 0.8) | 0.005 | 0.8 (0.1 - 4.5) | 0.76 |
| Alcoholic | | | | | | |
| Yes | 61 (12%) | 27 (16%) | 1.2 (0.7 - 2.0) | 0.43 | 0.8 (0.4 - 1.6) | 0.60 |
| No | 313 (60%) | 114 (68%) | 1.00 |  | 1.00 |  |
| NA* | 149 (29%) | 27 (16%) | 0.5 (0.3 - 0.8) | 0.005 |  | |
| TB Contact (Outside Household) | | | | | | |
| Yes | 35 (7%) | 18 (11%) | 1.7 (0.9 - 2.9) | 0.08 | 1.6 (0.9 - 3.0) | 0.11 |
| No | 488 (93%) | 150 (89%) | 1.00 |  | 1.00 |  |
| Sleeping with Index (After Diagnosis) | | | | | | |
| Same room, same bed | 157 (30%) | 50 (30%) | 1.00 |  | 1.00 |  |
| Same room, diff. bed | 217 (42%) | 70 (42%) | 1.0 (0.7 - 1.6) | 0.95 | 1.1 (0.6 - 1.8) | 0.82 |
| Same house, diff. room | 144 (28%) | 44 (26%) | 1.0 (0.6 - 1.5) | 0.86 | 1.1 (0.6 - 1.8) | 0.82 |
| Others | 5 (1%) | 4 (2%) | 2.5 (0.9 - 9.9) | 0.19 | 2.5 (0.5 - 11.8) | 0.24 |
| INDEX Cavity on CXR | | | | | | |
| Present | 238 (49.4%) | 81 (56%) | 1.3 (0.8 - 1.9) | 0.25 | 1.2 (0.8 - 1.8) | 0.42 |
| Absent | 244 (50.6%) | 65 (45%) | 1.00 |  | 1.00 |  |
| Smear & Culture | | | | | | |
| C- S- | 75 (14%) | 20 (12%) | 1.00 |  | 1.00 |  |
| C- S+ | 5 (1%) | 1 (0.6%) | 0.8 (0.2 - 3.5) | 0.71 | 0.7 (0.2 - 2.7) | 0.59 |
| C+ S- | 142 (27%) | 45 (27%) | 1.2 (0.7 - 2.1) | 0.56 | 1.8 (0.9 - 3.4) | 0.01 |
| C+ S+ | 301 (58%) | 102 (61%) | 1.3 (0.8 - 2.1) | 0.37 | 1.5 (0.8 - 2.8) | 0.19 |

^*^Not applicable in Alcoholic omitted because of collinearity. Odds ratios were adjusted for Household cluster.
