# Supplementary material for: Tuberculin skin test and QuantiFERON-Gold In Tube assay for diagnosis of latent TB infection among household contacts of pulmonary TB patients in high TB burden setting
Source: PLoS One. 2018 Aug 1;13(8):e0199360. doi: 10.1371/journal.pone.0199360 (PMC6070176; doi:10.1371/journal.pone.0199360)
Supplement: S5 Table — (DOCX) [file pone.0199360.s005.docx]

**S5: Table showing response to TST and QFT-GIT among those who had BCG vaccination**

| Test | BCG Scar | | Total | Sig. |
| --- | --- | --- | --- | --- |
|  | Present | Absent |  |  |
| QFT-GIT | | | | |
| Pos. | 260 (53.8%) | 208 (53.9%) | 468 | >0.950 |
| Neg. | 223 (46.2%) | 178 (46.1%) | 401 |  |
| TST(≥5mm) | | | | |
| Pos. | 248 (51.3%) | 230 (59.6%) | 478 | 0.016 |
| Neg. | 235 (48.7%) | 156 (40.4%) | 391 |  |
| LTBI* | | | | |
| Pos. | 343 (71.0%) | 303 (78.5%) | 646 | 0.012 |
| Neg. | 140 (29.0%) | 83 (21.5%) | 223 |  |
| TST(≥10mm) | | | | |
| Pos. | 133 (27.5%) | 95 (24.6%) | 228 | 0.352 |
| Neg. | 350 (72.5%) | 291 (75.4%) | 641 |  |
| LTBI** | | | | |
| Pos. | 287 (59.4%) | 230 (59.6%) | 517 | >0.950 |
| Neg. | 196 (40.6%) | 156 (40.4%) | 352 |  |

*= positive by either by QFT-GIT or by TST >5mm

**= positive by either by QFT-GIT or by TST >10mm
