# Supplementary material for: Tuberculin skin test and QuantiFERON-Gold In Tube assay for diagnosis of latent TB infection among household contacts of pulmonary TB patients in high TB burden setting
Source: PLoS One. 2018 Aug 1;13(8):e0199360. doi: 10.1371/journal.pone.0199360 (PMC6070176; doi:10.1371/journal.pone.0199360)
Supplement: S6 Table — (DOCX) [file pone.0199360.s006.docx]

**S6: Sensitivity and Specificity of LTBI by Latent Class analysis method (with TST ≥10mm)**

| Test | Sensitivity | Specificity |
| --- | --- | --- |
| TST (T) ≥10mm | 99% | 42% |
|  | (79% - 100%) | (20% - 67%) |
| QFT-GIT (Q) | 88% | 79% |
|  | (76% - 100%) | (73% - 84%) |
| Exposure (E) | 38% | 71% |
|  | (31% - 45%) | (64% - 76%) |
|  | | |

Estimated Prevalence = 63.0% (25.9% - 88.9%)

Note: Probability of being LTBI Positive: T+Q+E+ (100%), T+Q+E- (99.9%), T+Q-E+ (99.8%), T+Q-E- (99.7%), T-Q+E+ (88.1%), T-Q+E- (83.6%), T-Q-E+ (21.5%) and T-Q-E- (15.8%).
