# Supplementary material for: Tuberculin skin test and QuantiFERON-Gold In Tube assay for diagnosis of latent TB infection among household contacts of pulmonary TB patients in high TB burden setting
Source: PLoS One. 2018 Aug 1;13(8):e0199360. doi: 10.1371/journal.pone.0199360 (PMC6070176; doi:10.1371/journal.pone.0199360)
Supplement: S7 Table — (DOCX) [file pone.0199360.s007.docx]

**S7 Table: Consolidated Table of Data Pattern and Probability of LTBI by Latent Class analysis method**

| TST | QGIT | Exposure | TST ≥10mm | | TST ≥5mm | |
| --- | --- | --- | --- | --- | --- | --- |
|  |  |  | n (%) | p(LTBI+_ve_) | n (%) | p(LTBI+_ve_) |
| Pos | Pos | High | 123 (14.2%) | 100.0% | 212 (24.4%) | 93.5% |
| Pos | Pos | Low | 56 (6.4%) | 99.9% | 88 (10.1%) | 90.2% |
| Pos | Neg | High | 38 (4.4%) | 99.8% | 122 (14.0%) | 74.3% |
| Pos | Neg | Low | 11 (1.3%) | 99.7% | 56 (6.4%) | 64.8% |
| Neg | Pos | High | 201 (23.1%) | 88.1% | 112 (12.9%) | 56.3% |
| Neg | Pos | Low | 88 (10.1%) | 83.6% | 56 (6.4%) | 45.1% |
| Neg | Neg | High | 225 (25.9%) | 21.5% | 141 (16.2%) | 20.4% |
| Neg | Neg | Low | 127 (14.6%) | 15.8% | 82 (9.4%) | 14.1% |
